# Substitution of the CD81 Binding Site and β-Sandwich Area in E2 of HCV in Cambodia

**DOI:** 10.3390/v12050551

**Published:** 2020-05-16

**Authors:** Chikako Yamamoto, Shintaro Nagashima, Channarena Chuon, Ko Ko, Son Huy Do, Oline Lim, Sirany Hok, Somana Svay, Junko Matsuo, Keiko Katayama, Kazuaki Takahashi, Junko Tanaka

**Affiliations:** 1 Department of Epidemiology, Infectious Disease Control and Prevention, Hiroshima University Graduate School of Biomedical and Health Science, 1-2-3, Kasumi, Minami-ku, Hiroshima-shi 734-8551, Japan; c-yamamoto@hiroshima-u.ac.jp (C.Y.); s-nagashima@hiroshima-u.ac.jp (S.N.); chuonchannarena@rocketmail.com (C.C.); d161800@hiroshima-u.ac.jp (K.K.); matsujunn@hiroshima-u.ac.jp (J.M.); keikata@hiroshima-u.ac.jp (K.K.); ktakaha@hiroshima-u.ac.jp (K.T.); 2 Department of Health, Binh Thuan Medical College, Binh Thuan Province, 274 Nguyen Hoi Street, Phan Thiet City 800000, Vietnam; huyson68@gmail.com; 3 Ministry of Health, Phnom Penh, No: 80, Samdach Penn Nouth Blvd (289), Sankat Beoungkak 2, Tuol Kork District, Phnom Penh 12152, Cambodia; olinelim@yahoo.com (O.L.); hoksirany@yahoo.com (S.H.); 4 University of Health Sciences, Phnom Penh, #73, Preath Moniving blvd, Sangkat Sras Chak Khan Daun Penh, Phnom Penh 12201, Cambodia; somana53a@gmail.com

**Keywords:** hepatitis C virus, Cambodia, general population, mutation rate, CD81 binding site

## Abstract

The high genetic variability of hepatitis C virus (HCV) is the main obstacle to developing a vaccine. E2 has attracted attention for vaccine development because targeting this protein could potentially overcome issues related to the genetic diversity of HCV. In this study, we analyzed HCV genes in the general population of Cambodia and investigated the E2 locus as a candidate for vaccine development. HCV sero-epidemiological surveys were conducted between the period 2010 and 2014, with an HCV RNA–positive rate of 1.3% (11/868). Follow-up blood samples were collected from four anti-HCV– and HCV RNA– positive patients (genotype 1b: 2 cases, 6e: 1 case, 6r: 1 case) after 4.12 years. Analysis of HCV full-length nucleotide sequences in paired specimens revealed that the mutation rates of HCV genotypes 1b and 6e/6r were 1.61–2.03 × 10^−3^ and 2.52–2.74 × 10^−3^ substitutions/site/year, respectively. Non-synonymous substitutions were detected in HVR1, the front layer of the CD81 binding site, and the β-sandwich, but not in the N-terminal region or adjacent to the CD81 binding site. Therefore, we conclude that the CD81 binding site is a promising locus for HCV vaccine development.

## 1. Introduction

An estimated 71 million people have chronic hepatitis C virus (HCV) infection, and approximately 399,000 die from HCV each year [1]. In some countries, there is a deficit of information about the prevalence and genetic mutation of HCV [2].

HCV is a small, enveloped, positive-sense single-stranded ribonucleic acid (RNA) virus belonging to genus *Hepacivirus*, a member of family *Flaviviridae* [3]. The gene sequence of HCV is diversified by an error-prone polymerase that does not have a proofreading function during replication [4]. HCV strains are classified into seven genotypes (1–7). The distribution of HCV genotypes differs by country and region [5]. Genotype 1b is the most common globally (46%) [2], whereas genotype 6 is predominant in Southeast Asia [6]. In fact, the distribution of HCV genotypes is closely related to the pathway of virus transmission and human migration [7]. Therefore, the characterization of HCV genotypes correlates with both clinical features (natural history and therapy) and epidemiology [8]. 

HCV quasispecies are generated by synonymous substitution and nonsynonymous substitution [4]. The ratio of nonsynonymous to synonymous substitutions (dN/dS) reflects the relative immune pressure at a given locus [9]. 

The HCV envelope glycoproteins E1 and E2 mediate the entry of the virus, and the envelope region has drawn attention as a potential vaccine target that could overcome challenges related to the genetic diversity of HCV [10,11,12]. Although direct-acting antivirals (DAAs) with a high sustained virologic response (SVR) rate have been developed for HCV, the availability of DAAs is limited due to their high cost [13]; moreover, drug-resistant virus may develop, and patients remain vulnerable to reinfection after cure [14].

Once an HCV vaccine is developed, it would be useful in children born to HCV-infected mothers and health care workers who have frequent exposure to blood and body fluids, as well as in many countries that have high HCV prevalence [15]. 

E2 is the main target of neutralizing antibodies. It interacts with the low-density lipoprotein (LDL), scavenger receptor class B (SR-BI), CD81, and other cell surface molecules to mediate virus entry [14]. In particular, CD81 is exploited by genotypes 1a, 1b, 2a, 2b, 3a, 4, 5, and 6 for cell entry. Amino acids 483–499 of E2 constitute a linear epitope that forms the outer layer of the β-sandwich on the HCV surface; this epitope binds to the antibody as a part of an antigen group [16]. Olbrich et al. [16] predicted that an effective HCV vaccine could be developed if it were possible to target the neutralizing antibody reaction against the β-sandwich part of HCV.

Cambodia has high age-standardized mortality from liver cancer, 21.9/100,000 population [17]. Hepatitis virus infections, especially HBV and HCV, are leading causes of HCC. However, there is limited information about the sero-epidemiology and molecular epidemiology of HBV and HCV infections in Cambodia. Since 2009, we have conducted epidemiological studies in cooperation with the Ministry of Health of Cambodia [18,19,20,21,22]. Because samples from previous surveys were preserved in our laboratory, it was possible to analyze the genetic sequences of HCV from Cambodia. In this study, we investigated the locus at E2 among the general population in Cambodia as a potential target for the development of an HCV vaccine.

## 2. Materials and Methods

### 2.1. Subjects

The sero-epidemiological studies were conducted in the general population in Siem Reap, Cambodia from 2010 until 2014. Subjects ranged in age from 7 to 90 years.

### 2.2. Ethical Issues

This study was approved by the Ethics Committee for Epidemiological Research of Hiroshima University (No.223-2, approval date: 14 March 2016) and the Ministry of Health of Cambodia (ethical No. 0085NECH, approval date: 6 June 2013). Written informed consent was obtained from all participants. For subjects under the age of 18 years, consent was obtained from a parent or legal guardian before samples were collected. All research was performed in accordance with relevant guidelines and regulations. 

### 2.3. Serological Tests

Approximately 10 mL of whole blood was centrifuged. After centrifugation, sera were kept at −30 °C and transferred to Hiroshima University. HCV infection was assessed as follows. All sera were tested for HCV antibody (anti-HCV) and HCV RNA. Anti-HCV was measured using the particle agglutination test (PA) (Ortho HCV Ab PA test II; Ortho-Clinical Diagnostics, Tokyo, Japan). HCV infection was defined as seropositivity for HCV antibody. HCV RNA was extracted from 100 μL of anti-HCV positive sera using SMITEST EX-R & D (Genome Science Laboratories, Fukushima, Japan). HCV RNA was subjected to real-time PCR using primers targeting the 5’ non-coding region (Supplementary Table S1). RT-PCR conditions were as follows: reverse transcription reaction at 50 °C for 5 min, initial activation at 95 °C for 20 s, and 55 cycles of denaturation at 95 °C for 5 s and annealing and extension at 60 °C for 40 s. Reactions were performed on an StepOne Real-Time PCR System (Applied Biosystems/Thermo Fisher Scientific, Waltham, MA, USA).

### 2.4. Genotype Analysis

RT-PCR was performed on the HCV core region using PrimeScript High Fidelity One Step RT-PCR Kit (Takara Bio Inc., Kusatsu, Shiga, Japan). Total 10 primers (Supplementary Table S2) were used for HCV core region sequence analysis. RT-PCR conditions were as follows: reverse transcription at 45 °C for 10 min; initial activation at 94 °C for 2 min; and 35 cycles of denaturation at 98 °C for 10 s, annealing at 55 °C for 15 s, and elongation at 68 °C for 20 s. Reactions were performed on a GeneAmp PCR System 9700 (Applied Biosystems/Thermo Fisher Scientific, Tokyo, Japan). PrimeStar GXL DNA Polymerase Kit (Takara Bio) was used for secondary PCR (2nd PCR). Conditions for 2nd PCR were as follows: 30 cycles of denaturation at 98 °C for 10 s, annealing at 55 °C for 15 s, and extension at 68 °C for 3 min. PCR products were sequenced using the Big-Dye Terminator v3.1 Cycle Sequencing Kit (Thermo Fisher Scientific, Tokyo, Japan) and defined using a 3730 xl DNA Analyzer (Applied Biosystems/Thermo Fisher Scientific). For HCV RNA–positive cases, HCV genotype was determined by phylogenetic tree analysis of the core region using the neighbor-joining method (NJ method) [23] in MEGA ver. 7 [24]. 

### 2.5. HCV Full-Length Genome Sequence Analysis

Primers were designed to overlap more than 10 regions (Supplementary Tables S3–S5) based on the sequences of known isolates of genotype 1b, 6e, or 6r registered in GenBank. PrimeScript High Fidelity One Step RT-PCR Kit (Takara Bio) was used for RT-PCR. 

Cycling was performed as described above for RT-PCR of the core region, except that extension time was 30 s. For 2nd PCR, the composition of the sample and the PCR conditions were as described above for sequence analysis of the core region.

### 2.6. Phylogenetic Tree Analysis

The sequence information of known strains closely related to those examined in this study was obtained from the site of National Center for Biotechnology Information (NCBI), and a phylogenetic tree was constructed using the Minimum Evolution method [25] in MEGA ver-7 [24].

### 2.7. Calculation of HCV Mutation Rate in the Same Individuals of Cambodia

For four HCV RNA–positive subjects, a second round of samples were taken 4.12 years after the original sample collection. An HCV full-length genome sequence was compared between samples collected in 2012 and 2016. HCV mutation rate was defined as substitutions/site/year [26]. Data were analyzed using JMP version 11 (SAS Institute, Cary, NC, USA). Furthermore, the HCV genotype mutation rates of HCV genotype 1b and genotype 6 (6e, 6r) were compared using the Bonferroni correction of the χ^2^ test.

### 2.8. Estimation of Mutation in Each Genetic Region of HCV Sampled from the Same Cambodian Subjects

The ratio of nonsynonymous substitution rate to synonymous substitution rate (dN/dS) was investigated as an indicator of immune pressure in each region of HCV RNA in the four individuals mentioned above [27]. Calculation of nonsynonymous substitution, synonymous substitution, and immunological pressure was conducted in Genetic Mac ver-7.

### 2.9. Nonsynonymous Substitution at E2 in the Same Cambodian Subject

Nonsynonymous substitution in a single Cambodian subject was investigated at HVR1 (amino acids 384–410), HVR2 (amino acids 474–480) [28], three regions of the CD81 binding site [18,29], and the β-sandwich area (amino acids 483–499).

## 3. Results

A total of 868 subjects (360 [41.5%] male and 508 [58.5%] female) from Cambodia participated in this study.

### 3.1. Serologic Analysis

In Cambodia, the prevalence of anti-HCV was 3.9% (34/868; 95% Confidence Interval [CI], 2.6–5.2%), and the prevalence of HCV RNA was 1.3% (11/868; 95% CI, 0.55–2.1%]). 

### 3.2. HCV Genotype

**Figure 1:**
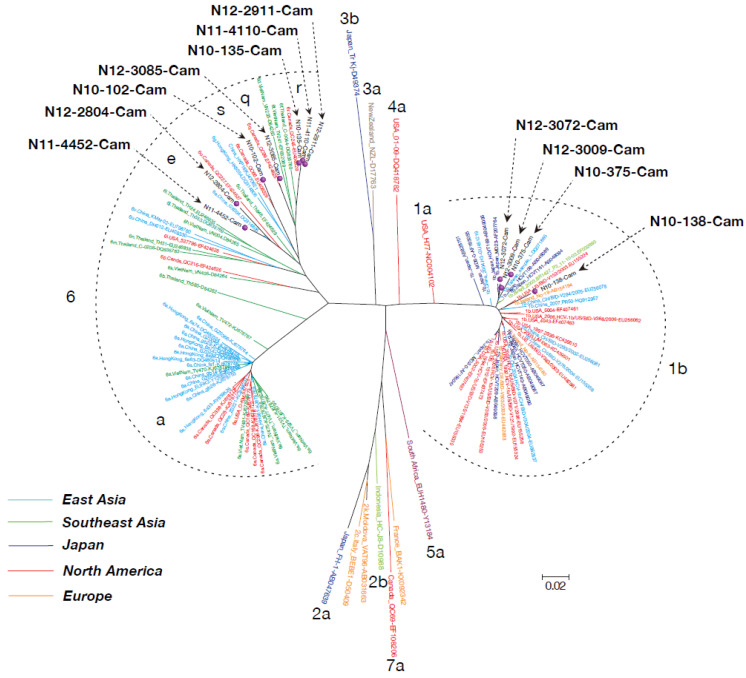
Phylogenetic analysis based on the core region of hepatitis C virus (HCV) RNA in carriers from the general population of Cambodia. The phylogenetic tree was constructed using the neighbor-joining method. The subtypes (1a~7a) are indicated near the each HCV isolates. The 11 genomes characterized in this study are indicated with a red circle.

Among 11 HCV RNA–positive cases, four (36.4%) were identified as genotype 1b, and seven (63.6%) as genotype 6 (two 6e, three 6r, one 6q, and one 6s) (Figure 1).

### 3.3. Phylogenetic Tree Analysis of HCV Near Full-Length Genome Sequence

We performed nearly full-length genome sequence analysis on eight cases (Cambodia 1b: four cases, 6e: two cases, and 6r: two cases) (Table 1). Next, we obtained nearly full or full genome sequence of genotype 1b (282 known isolates) from around the world and phylogenetically analyzed four cases (Cambodia isolate: N12-3009-Cam, N16-3009-Cam, N12-3072-Cam, and N16-3072-Cam).

**Figure 2:**
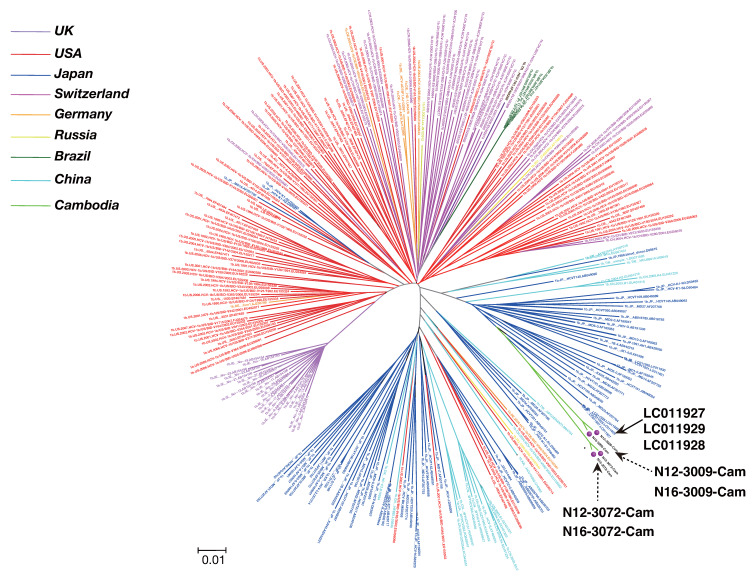
Phylogenetic trees of HCV genotype 1b near full-genome sequence. GenBank accession numbers are shown in parentheses; scale bar indicates nucleotide substitutions per site. The four genomes characterized in this study are indicated with red circles.

The genotype 1b isolate from our Cambodia study was closely related to Japanese strains LC011927, LC011929, and LC011928, with a nucleotide identity of 92.53–92.43%. (Figure 2). Nearly full or full genome sequences of genotype 6 (250 known isolates) from around the world and four cases from our study (Cambodia isolates: N12-2804-Cam, N16-2804-Cam, N12-2911-Cam, and N16-2911-Cam) were subjected to phylogenetic tree analysis.

Phylogenetic tree constructed based on HCV genotype 1b. The phylogenetic tree was constructed by the Minimum Evolution method using 282 previously reported isolates.

**Figure 3:**
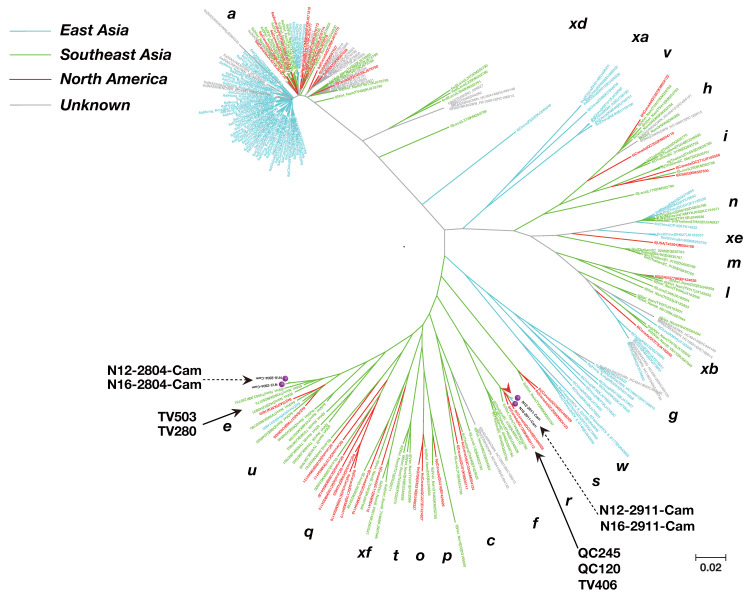
Phylogenetic tree constructed based on HCV genotype 6 near full-genome sequence. The phylogenetic tree was constructed using the Minimum Evolution method using 250 previously reported isolates. The subtypes (a~xf) are indicated near the each HCV isolates. The four genomes characterized in this study are indicated with red circles.

The genotype 6e isolate from our Cambodia study was closely related to strains from Vietnam (TV 503 and TV 280) and the United States (P), with a nucleotide sequence identity of 85.76–92.27%. The genotype 6r isolates (N12-2911-Cam and N16-2911-Cam) from our study were closely related to strains from Asian immigrants living in Canada (QC245 and QC120) and Vietnamese strain TV 406, with a nucleotide sequence identity of 90.13–96.38% (Figure 3).

### 3.4. Mutation Rate of HCV Genotypes 1b and 6

**Figure 4:**
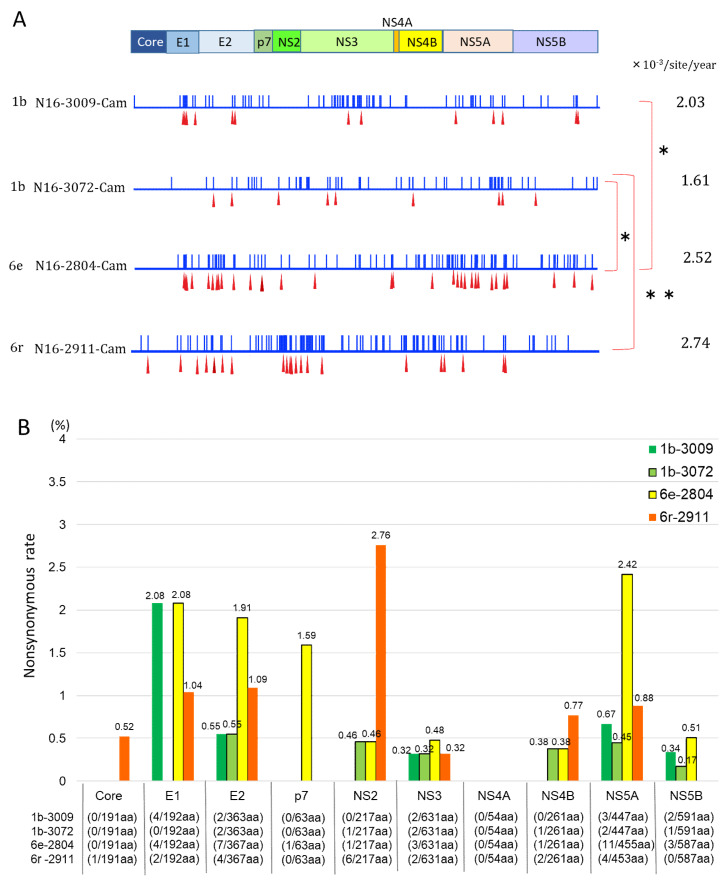
Mutation sites and the rate of nonsynonymous substitution of HCV RNA. (**A**) Nucleotide and amino acid mutation sites of HCV RNA among four Cambodian subjects. Blue bar indicates nucleotide mutation; red triangles indicate amino acid mutations. A significant difference (*, **) in mutation rate was observed between genotypes 1b (No.3009) and 6e (No.2804) (*p* < 0.0001); 1b (No.3009) and 6r (No.2911) (*p* = 0.042); 1b (No.3072) and 6e (No.2804) (*p* < 0.0001); and 1b (No.3072) and 6r (No.2911) (*p* = 0.00070). However, there was no significant difference between genotypes 1b (No.3009) and 1b (No.3072) (*p* = 0.18) or 6e (No.2804) and 6r (No.2911) (*p* = 0.55). (**B**) The rate of nonsynonymous substitution in each area of HCV RNA in the same four Cambodian subjects. The numbers of nonsynonymous substitutions per area of HCV RNA are described in each of 10 areas.

We calculated the mutation rate of HCV RNA based on the number of mutated base pairs in samples collected 4.12 years apart from four cases in Cambodia. Expressed as substitutions/site/year, the mutation rates were 2.03 × 10^−3^ (95% CI: 1.58 × 10^−3^–2.48 × 10^−3^) in genotype 1b (No. 3009), 1.61 × 10^−3^ (95% CI: 1.21 × 10^−3^–2.02 × 10^−3^) in genotype 1b (No. 3072), 2.52 × 10^−3^ (95% CI: 2.03 × 10^−3^–3.04 × 10^−3^) in genotype 6e (No. 2804), and 2.74 × 10^−3^ (95% CI: 2.23 × 10^−3^–3.26 × 10^−3^) in genotype 6r (No. 2911) (Figure 4A). 

### 3.5. Nonsynonymous Substitution Rate and Immune Pressure in the HCV RNA

**Figure 5:**
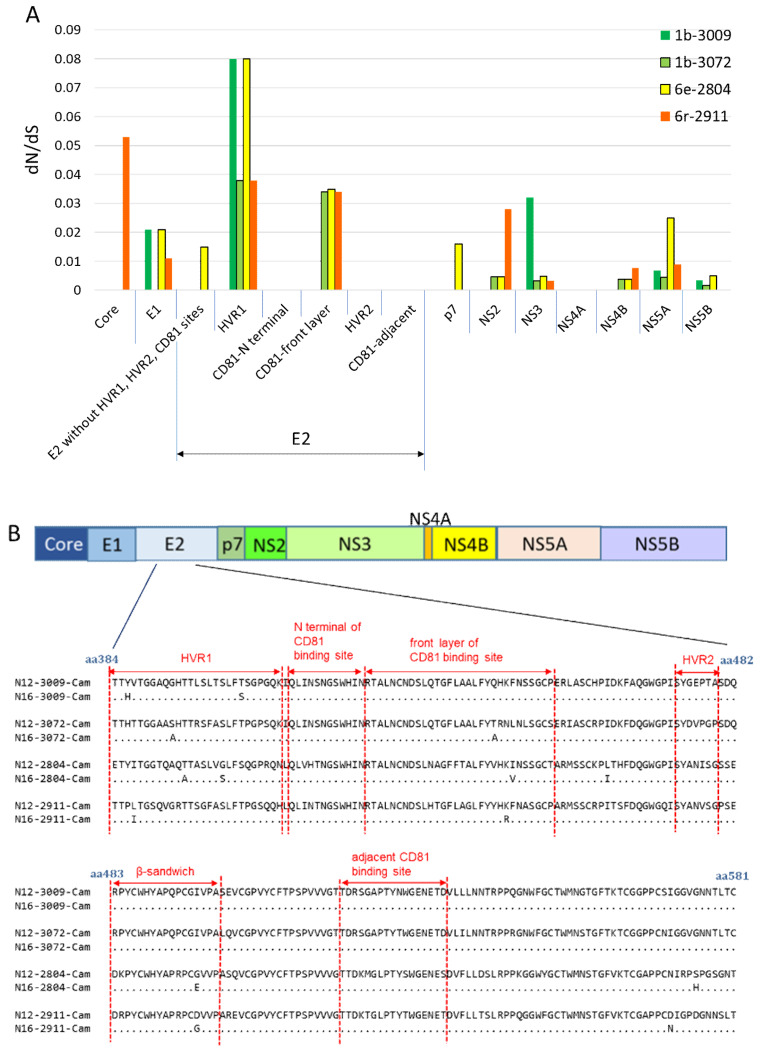
Immune pressure on HCV RNA and amino acid mutation of E2 (**A**) Immune pressure in each area of HCV RNA among four Cambodian subjects Ten areas of HCV RNA and six loci in E2 are described. (**B**) Amino acid mutation of the HVR, CD81 binding site (CD81bs), and β-sandwich in E2 over 4.12 years. The amino acid sequence at E2 at two points among the four subjects is indicated. Red dotted lines indicate HVR1 (aa 384–410), HVR2 (aa 474–480), N-terminus of CD81bs (aa 412–423), the front layer of CD81bs (aa 424–453), the adjacent CD81-binding loop (aa 519–535), and the β-sandwich (amino acids 483–499). Of the three regions of the CD81-binding site, nonsynonymous substitution occurred only at the front layer in 1b (No.3072), 6e (No.2804), and 6r (No.2911), whereas in the remaining two regions (N-terminal and adjacent), no nonsynonymous substitution was observed (Figure 5B).

Common nonsynonymous substitutions in E2 occurred in all four subjects. The nonsynonymous substitution rates were 0.55% in 1b (No.3009) and 1b (No.3072), 1.91% in 6e (No.2804), and 1.09% in 6r (No.2911) (Figure 4B). Within the E2 locus, nonsynonymous substitution predominated in HVR1, but not in HVR2. In these four subjects, the gene locus exhibiting the highest immune pressure was HVR1: dN/dS was 0.080 for 1b (No. 3009) and 6e and 0.038 for 1b (No. 3072) and 6r (Figure 5A).

Focusing on the β-sandwich region, genotypes 1b, 6e, and 6r had different amino acid sequences. Over the elapsed time of 4.12 years, no amino acid mutations occurred in the β-sandwich sequence of genotype 1b, whereas mutation was observed in genotypes 6e and 6r (Figure 5B).

## 4. Discussion

Among 868 Cambodian subjects, the prevalence of anti-HCV antibodies was 3.9%, and the prevalence of HCV RNA positivity was 1.3%. The former value is lower than the previously reported prevalence of anti-HCV antibodies among Cambodians, 6.5–8.0% [30,31]. 

HCV genotypes 1a, 1b, 2a, and 3a are classified global epidemic strains [32]; consistent with this, they are distributed all over the world, especially in high-income countries [33]. The strains were reportedly spread to various countries during and after World War II [34], as well as by blood transfusion and intravenous drug use in the 1970s and 1980s [33]. In addition, the prevalence of HCV in soldiers returning to the United States after the Vietnam War was 10–17%, higher than the prevalence of 1.3% in North American residents overall [35,36]. There are the reports that HCV genotype 1b was spread worldwide through World War II [37]. 

On the other hand, HCV genotype 6, classified as a local epidemic strain, is predominant in Southeast Asia [32]. Phylogenetic tree analysis revealed that genotypes 6e and 6r in Cambodia are closely related to strains from Asian immigrants living in the United States and Canada. Consistent with this, genotype 6 is commonly found [38] in immigrants from Southeast Asia to the United States and Canada. Genotype 6 is thought to have originated in Southeast Asia [37].

The HCV RNA consists of 9600 bases, and it is not easy to perform full-length gene analysis [39]. This was the first study to investigate the nonsynonymous substitution rate and mutation rate of each gene region by performing full genome sequencing analysis of HCV genotypes 1b, 6e, and 6r from the general population.

Previous studies reported that the full-genome mutation rate in HCV patients is 1.2–1.92 × 10^−3^ substitutions/site/year [27,40], not significantly different from the mutation rate measured in this study. It is conceivable that the number of nonsynonymous substitutions in the full RNA was 2–3-fold greater in genotypes 6e and 6r than in genotype 1b due to a higher mutation rate in the former strains. Among all genotypes, genotype 6 has the largest number of subtypes (29 subtypes, 6a–6xf) [8]; this could be because it is one of the oldest genotypes [41], but also potentially due to its high mutation rate. In this study, the mutation rate of HCV could only be studied in four cases, limiting opportunities for comparison. 

In all 11 HCV genes, the rate and speed of synonymous substitution were higher than those of nonsynonymous substitution. A previous study reported results consistent with our findings [42]. Another study reported that genetic diversity is not evenly distributed throughout the HCV genome, and that HVR1 at the 5’ end of the E2 region is the most diverse [43]. We also observed the highest variability at HVR1, indicating that immune pressure is greatest at this locus. The hyper-variable region 1 (HVR1), a small fragment spanning 27 amino acids of E2 on highly variable region of HCV genome, is a sequence mutation that plays a role in evading neutralization by HCV-specific antibodies [44]. We assume that nonsynonymous substitutions are related to immunologic escape, whereas synonymous substitutions are not the direct consequence of immunological events. HVR1, with the highest immune pressure, serves as an “immunologic decoy” [27], ensuring that HCV is not neutralized. HCV enters the cell after binding cell surface receptors such as CD81. Therefore, E2 is considered to be the primary target of the neutralizing antibody reaction [45]. E2 has attracted a great deal of attention as an important site for HCV vaccine development [29,45,46], but this locus contains a hypervariable region with the highest genetic diversity in the viral genome. Consequently, HCV vaccine development is difficult. Because HVR1 is the surface antigen of the virus, it plays an important role in evading the host immune response [47]. Through HVR1 mutation, HCV evades the host’s neutralizing antibodies and causes persistent infection [26]. The mutation rate of HVR1 over 4.12 years was extremely high in all four cases characterized in this study. Another study reported that the mutation rate of HVR1 in HCV patients is the highest in the HCV RNA genome [48]. Because E2 is structurally flexible, HVR1 can be removed without affecting the structure of the glycoprotein and the removal of HVR1 facilitates binding of HCV to CD81 [46].

An epitope produced by an HCV strain harboring an HVR1 deletion and E2 mutation was used to immunize mice, which subsequently produced an antibody that bound strongly to CD81; however, cross-neutralization did not occur [45].

HCV binds to CD81 when entering hepatocytes [49]. Therefore, if a vaccine could be developed targeting the CD81 binding region of HCV, it would be effective in preventing infection by diverse genotypes. Of the three regions in the CD81 binding site (front layer, N-terminal region, and adjacent CD81 binding loop) [29], the nucleotide sequence conservation of two regions (N-terminal and adjacent) was high after 4.12 years. Since we compared HCV mutations among only four persons, it is limited to identify CD81 binding site as the promising locus for HCV vaccine development in this study. According the previous report [50] about CD81 binding site, they focused on the reaction to multiple bNAbs and structural features, suggested that CD81 binding site may be useful for HCV vaccine development. 

The binding of HCV E2 proteins to CD81 on NK cells was shown to be associated with an impaired NK cell-mediated cytolytic function and an impaired IFN-gamma production [51]. The future efforts aimed at HCV vaccine development research should focus on the CD81 binding site.

Mutations in the sequence of the β-sandwich area in E2 after 4.12 years have not yet been compared between genotypes 1b, 6e, and 6r. However, we found that genotypes 1b, 6e, and 6r have different amino acid sequences in the β-sandwich. There are some previous reports about HCV vaccine development focusing on the HCV β-sandwich [52]. Consequently, if the β-sandwich is targeted, it may be necessary to develop a particular vaccine for each genotype.

The potential application of HCV proteins to develop vaccines, and especially the use of precise epitopes of structural proteins or various linear or conformational epitopes of core, E1, E2, NS3 and NS4 and different T-cell stimulating epitopes of core, E1, E2, and NS3 regions as immunizing agents, have been disclosed [53,54,55,56].

Some barriers to HCV vaccine development derived from the features of HCV are reported to be due to (1) An HCV genomic variability with seven distinct genotypes with more than 65 subtypes which differ in nucleotide sequence, (2) a high error-prone mutation rate of HCV with the ability to escape selection pressure by nAbs and CD8^+^ T cells, (3) a high mutation rate occurring in the HVR 1 of E2 along with the potential of HVR1 to interfere with the binding of antibodies to E2, (4) the cell-to-cell transmission of HCV constituting a considerable hindrance to developing a B-cell-based HCV vaccines that induces broad cross-nAb, since HCV could avoid the extracellular component.

On the other hand, the other feature of HCV may be useful for HCV vaccine development.

Three major approaches have been adopted for vaccine design against HCV. The traditional approach uses recombinant envelope proteins to induce neutralizing antibodies (nAb) [56,57]. The second approach uses virus-like particles (VLPs) that express HCV structural proteins to induce both humoral and cellular immunity [58,59]. The third and most promising approach is designing an HCV vaccine that would induce a potent T cell immune response [56]. The selection of antigens that maximize the induction of T-cell and antibody responses that elicit successful responses remains an active area of research.

Furthermore, HCV in circulation binding to plasma lipoprotein to form an infectious hybrid lipoviral particle (LVP) that promotes viral persistence and a high infection by limiting the access of nAbs to envelope glycoprotein are factors that pose a significant challenge to developing an effective HCV vaccine [60]. 

An HCV vaccine that can generate cross-nAbs and cell-mediated immune responses should be the goal. Two vaccines targeting the antibody or T-cell responses are currently in preclinical or clinical trials. Next-generation vaccines will likely involve a combination of these two strategies.

## Figures and Tables

**Table 1:** Characteristics of positive samples collected from Cambodia.

Genotype	Isolate Name	Nucleotide Length	Polyprotein Length	Blood Sampling Date	Country	Province	Village	Sex	Age	Occupation
1b	N12-3009-Cam	9323	3010	2012.08.22	Cambodia	Siem Reap	Krabei Riel	male	60s	teacher
	N16-3009-Cam	9326	3010	2016.09.03						
	N12-3072-Cam	9378	3010	2012.08.22	Cambodia	Siem Reap	Chrey	female	70s	housewife
	N16-3072-Cam	9321	3010	2016.09.03						
6e	N12-2804-Cam	9341	3018	2012.08.22	Cambodia	Siem Reap	Krabei Riel	male	20s	office worker
	N16-2804-Cam	9326	3018	2016.09.03						
6r	N12-2911-Cam	9374	3016	2012.08.22	Cambodia	Siem Reap	Krabei Riel	female	60s	housewife
	N16-2911-Cam	9374	3016	2016.09.03						

This table shows the demographic characteristics of six positive samples collected from Cambodia between 2012 and 2016 that could be analyzed for full-length genome sequence.

## Supplementary Materials

The following are available online at https://www.mdpi.com/1999-4915/12/5/551/s1.
