# Supplementary material for: Substitution of the CD81 Binding Site and β-Sandwich Area in E2 of HCV in Cambodia
Source: Viruses. 2020 May 16;12(5):551. doi: 10.3390/v12050551 (PMC7290788; doi:10.3390/v12050551)
Supplement: Supplementary file 1 [file viruses-12-00551-s001.pdf]

Substitution of the CD81 Binding Site and β-Sandwich Area in E2 of HCV in Cambodia

Chikako Yamamoto [1], Shintaro Nagashima [1], Channarena Chuon [1], Ko Ko [1], Son Huy Do [2], Oline Lim [3], Sirany Hok [3], Somana Svay [4], Junko Matsuo [1], Keiko Katayama [1], Kazuaki Takahashi [1] and Junko Tanaka [1,*]

[1] Department of Epidemiology, Infectious Disease Control and Prevention, Hiroshima University Graduate School of Biomedical and Health Science, 1-2-3, Kasumi, Minami-ku, Hiroshima-shi 734-8551, Japan; c-yamamoto@hiroshima-u.ac.jp (C.Y.); s-nagashima@hiroshima-u.ac.jp (S.N.); chuonchannarena@rocketmail.com (C.C.); d161800@hiroshima-u.ac.jp (K.Ko.); matsujunn@hiroshima-u.ac.jp (J.M.); keikata@hiroshima-u.ac.jp (K.Ka.); ktakaha@hiroshima-u.ac.jp (K.T.)

[2] Binh Thuan Medical College, Binh Thuan Province, 274 Nguyen Hoi Street, Phan Thiet City, 800000, Vietnam; huyson68@gmail.com

[3] Ministry of Health, Phnom Penh, No: 80, Samdach Penn Nouth Blvd (289), Sankat Beoungkak 2, Tuol Kork District, Phnom Penh, 12152, Cambodia; olinelim@yahoo.com (L.O); hoksirany@yahoo.com (H.S)

[4] University of Health Sciences, Phnom Penh, #73, Preath Moniving blvd, Sangkat Sras Chak Khan Daun Penh, Phnom Penh, 12201, Cambodia; somana53a@gmail.com

* Correspondence: jun-tanaka@hiroshima-u.ac.jp; Tel: 082-257-5161, Fax: 082-257-5164

Supplementary Table Legends

Supplementary Table S1. The table show the primers used for 5′ non-coding region. sequence analysis of HCV.

Supplementary Table S1. Primers used for 5′ non-coding region sequence analysis.

**primer**	**Nucleotide sequences (5'-3')**
5'non-cod-forward	AGATTGCTAGCCGAGTAGTGTTG
5'non-cod-reverse	TGCACGGTCTACGAGACCTC
probe	Fam-AAGGCCTTGTGGTACTGCCTGATACGG-Tamra

Supplementary Table. 2. The table show the primers used for core region sequence analysis of HCV.

Supplementary Table S2. Primers for HCV core region sequence analysis.

**primer**	**Stage-polarity**	**Nucleotide sequences (5'-3')**
3s	sense	GGGAGAGCCATAGTGGTCTG
4s		GGAACCGGTGAGTACACCGG
4AS	antisense	CAACCRGGYARRTTCCCTGTTGC
5AS		GGAAGATAGARAAAGAGCAACC
6AS		AGAAGATAGAAAAGGAGCAACC
5s	sense	GCTAGCCGAGTAGYGTTGGGT
7s		GCGAAAGGCCTTGTGGTACTG
1AS	antisense	ATGTACCCCATGAGRTCGGC
2AS		ACGAGCGGRATGTACCCCATGAG
3AS		ACGACRGGGATGTACCCCATGAG

Supplementary Table S3. The table show the primers used for full genome sequence analysis of HCV genotype 1b. The primers were designed based on the known isolate: LC011927.

Supplementary Table S3. Primers used for full genome sequence analysis of HCV genotype 1b.

primer	Stage-polarity	Nucleotide sequences (5'-3')	Nt position
HCV-1-001	sense	GGGCGACACTCCACCATAGA	17-36
HCV-1-002	antisense	TAGTCGCGCGCACACCCAAC	38-57
HCV-1-003	sense	CACTCCCCTGTGAGGAACTA	470-489
HCV-1-004	antisense	GGCAACARGTAAACTCCACC	435-454
HCV-1-005	sense	TCGTGGAAGGCGACAACCTAT	515-535
HCV-1-006	antisense	AGAAGATAGAAAAGGAGCAACC	1459-1478
HCV-1-007	sense	GTAATYTGGGTAARGTCATCGA	691-712
HCV-1-008	antisense	GGAAGATAGARAAAGAGCAACC	1422-1443
HCV-1-009	sense	GGGAGAGCCATAGTGGTCTG	131-150
HCV-1-010	antisense	GCCCCCRCAYGTCTTGGTGAA	2019-2039
HCV-1-011		ACGGGGGGCCCCCRCAYGTCTT	2025-2046
HCV-1-012	sense	GGAACCGGTGAGTACACCGG	152-171
HCV-1-013	antisense	GTRCAGCCAAACCAGTTGCC	1980-1999
HCV-1-014		TCATCCAYGTRCAGCCRAACCA	1986-2007
HCV-1-015	sense	CCAGAGGCCYTAYTGYTGGCA	1784-1804
HCV-1-016	antisense	AARTATTGYRACCACCATAT	2868-2887
HCV-1-017	sense	TAYTGYTTCACCCCRAGCCC	1860-1879
HCV-1-018	antisense	ATAGTRYGGTGACARGGTCA	2827-2846
HCV-1-019	sense	TTCTGYGCYGCCTGGTACAT	2655-2674
HCV-1-020	antisense	TTTYTAYGGCGTRTGGCCGCT	2708-2728
HCV-1-021	sense	ACAGCRGCCCGGAAGATGCC	3873-3892
HCV-1-022	antisense	CTCGTGACCARGTAAAGGTC	3726-3745
HCV-1-023	sense	GGCGCCTAYGACATCATAAT	4263-4282
HCV-1-024	antisense	TCACCAGCCCGCTCACCACC	5731-5750
HCV-1-025	sense	TGTGATGARTGCCACTCAAC	4284-4303
HCV-1-026	sense	CATGTGGAAYTTCATCAGCG	5633-5652
HCV-1-027	antisense	ATGGAAGAGTGCGCCTCACAC	5463-5483
HCV-1-028	sense	TTCCGTGCCACGTGTTGCTA	6497-6516
HCV-1-029	antisense	AAGCAGAAGGCRCTCGGGTTG	5526-5546
HCV-1-030	sense	CGTTTTTGACATGTCCAGCGA	6445-6465
HCV-1-031	antisense	TGGGACTGGATATGCACGGT	6282-6301
HCV-1-032	sense	AAGGCGAAGGCGTCCACAGT	7812-7831
HCV-1-033	antisense	CCCATCAACGCRTACACCAC	6522-6541
HCV-1-034	sense	TACCGGGACGTGCTCAAGGA	7788-7807
HCV-1-035	antisense	TCCATGCCCCCCCTTGAGGG	7500-7519
HCV-1-036	antisense	ATCAATAGGGTGGCTTCATG	9042-9061
HCV-1-037	sense	AGCGACGGGTCTTGGTCTAC	7542-7561
HCV-1-038	antisense	TCACTCCATAGTTACTCTCCA	9015-9035
HCV-1-039	sense	GAGGCTATGACTAGGTACTC	8619-8638
HCV-1-040	antisense	CACTCCAGGCCAATAGGCCAT	9388-9408
HCV-1-041	sense	AGACAGCTAGACACACTCCA	8791-8810
HCV-1-042	antisense	TAAMCACTCCAGGCCAATAG	9384-9403
HCV-1-043	sense	TAAACTCACTCCAATCCCG	9200-9218
HCV-1-044	antisense	AAAAAAAAAAAAAAAAAAAAAAAAAA	9438-9463
HCV-1-045	sense	GGAGACATATATCACAGCG	9270-9288
HCV-1-044	antisense	AAAAAAAAAAAAAAAAAAAAAAAAAA	9438-9463
HCV-1-046	sense	ARRTACCTCTTCAAYTGGGC	9165-9184
HCV-1-047	antisense	AAAAAAAAAAAAAAAAAAAAAAAAAAAAAA	9438-9467
HCV-1-048	sense	ACNAAGCTMAAACTCACTCC	9192-9211
HCV-1-047	antisense	AAAAAAAAAAAAAAAAAAAAAAAAAAAAAA	9438-9467
HCV-1-049	sense	ACNAAGCTMAAACTCACTCC	9192-9211
HCV-1-043		TAAACTCACTCCAATCCCG	9200-9218
HCV-1-050	antisense	CGGCTCACGGACCTTTCACAG	9551-9571
HCV-1-051		TAGCCGTGACTAGGGCTAAG	9530-9549
HCV-1-045	sense	GGAGACATATATCACAGCG	9270-9288
HCV-1-052	antisense	TGGCCTGGAGTGGTTAGCTC	9380-9399
HCV-1-053		GAGTGGTTAGCTCCCCGT	9375-9392

Supplementary Table S4. The table show the primers (HCV-6-001~038) used for full genome sequence analysis of HCV genotype 6e. The primers were designed based on the known isolate: KM252780.

Supplementary Table S4. Primers used for full genome sequence analysis of HCV genotype 6e

primer	Stage-polarity	Nucleotide sequences (5'-3')	Nt position
HCV-6-001	sense	TTACGGGGCGACACTCCACC	10-29
HCV-6-002	antisense	TGCACGGTCTACGAGACCTC	831-853
HCV-6-003	sense	TCACTCCCCTKTGAGGAACTA	34-54
HCV-6-004	antisense	TATCAGGCAGTACCACAAGG	276-295
HCV-6-005	sense	GTCTAGCCATGGCGTTAGTA	74-93
HCV-6-006	antisense	ATCATCATRTCCCACGCCATG	1289-1309
HCV-6-007	sense	AGTACACCGGAATTGCCAGGA	159-179
HCV-6-008	antisense	CCYACCATGAGGTCCACATG	1119-1138
HCV-6-009	sense	GTAAGGTCATCGATACCCTT	697-716
HCV-6-010	antisense	ATCTCSACCCTATCACGATC	2316-2335
HCV-6-011	sense	GACCTCATGGGGTACATTCC	1125-1144
HCV-6-012	antisense	CAAGGRTARTGCCACAGCCT	2190-2209
HCV-6-013	sense	TGCTGGCACTACCCRCCAAGA	2193-2213
HCV-6-014	antisense	CTTCTCCATAGGCGAGAACA	3274-3293
HCV-6-015	sense	GTTGGTATGGGTGTACTTGG	1984-2003
HCV-6-016	antisense	ACAAATGCGCGCATCAGCTAG	2529-2549
HCV-6-017	sense	CTATGGCCACTCCTGCTGCT	2727-2746
HCV-6-018	antisense	GTGGAATGGCACTCGTCACA	4293-4312
HCV-6-019	sense	GGTGYCAGTATTTCATAGCTCG	2884-2905
HCV-6-020	antisense	CCTGTTGTTATRGTCCTGAC	4194-4213
HCV-6-021	sense	ATGTAYACYAATGTTGACCA	3648-3667
HCV-6-022	antisense	GCTGACATRCAGGTCATGAT	5283-5302
HCV-6-023	sense	GCCGTTTGTACCAGGGGAGT	3897-3916
HCV-6-024	antisense	CTTCCACATRTTGTCCCATGA	5157-5177
HCV-6-025	sense	GTCATAGACTGCAAYGTGGC	4701-4720
HCV-6-026	antisense	GGTGTTGTCCAATCCCCGTT	6243-6262
HCV-6-027	sense	TATCTCGCTGGCCTTTCCAC	5670-5689
HCV-6-028	antisense	TAATCYGGCCTYGCCCATAT	7212-7231
HCV-6-029	sense	CCTGCWTTAGAGGACATGGT	5970-5989
HCV-6-030	antisense	GCCACTGATATTTCCYTGTCGTC	7140-7162
HCV-6-031	sense	CTRACATCCATGCTRTCTGA	6861-6880
HCV-6-032	antisense	TTCTTGGAGCGCCACATCCG	8247-8266
HCV-6-033	sense	TCRGCYAGTCAGCTTTCTGC	6954-6973
HCV-6-034	antisense	AGGTGGTCCACGCGCTGCTTTG	8221-8242
HCV-6-035	sense	AACCACATCAACTCCGTGTG	7980-7999
HCV-6-036	antisense	AAGGAAGATGCCTACCCCTAC	9372-9392
HCV-6-037	sense	TGGCTAAGAATGAAGTGTTC	8047-8066
HCV-6-038	antisense	TAGGAGTAGGCACAGGAGTA	9346-9365

Supplementary Table S5. The table show the primers (HCV-6-039~102) used for full genome sequence analysis of HCV genotype 6r. The primers were designed based on the known isolate: EU408328.

Supplementary Table S5. Primers used for full genome sequence analysis of HCV genotype 6r

primer	Stage-polarity	Nucleotide sequences (5'-3')	Nt position
HCV-6-039	sense	TTACGGGGCGACACTCCACC	10-29
HCV-6-040	antisense	TGCACGGTCTACGAGACCTC	831-853
HCV-6-041	sense	TCACTCCCCTKTGAGGAACTA	34-54
HCV-6-042	antisense	TATCAGGCAGTACCACAAGG	735-757
HCV-6-043	sense	CCGGTTGCTCCTTTTCTATCT	847-867
HCV-6-044	antisense	CCGCACGTTTTGACGAAGCC	2016-2035
HCV-6-045	sense	TTGGCACTTCTTTCGTGCCT	873-892
HCV-6-046	antisense	GAGTTCATCCAGGTGCAGCC	1992-2011
HCV-6-047	sense	ATGGCGTCAGGGCCATCGAA	796-815
HCV-6-048	antisense	TCCGCGAGTCCAATTGCAAG	2787-2806
HCV-6-049	sense	AAGTGTGGGTCCCGAGTATG	2914-2933
HCV-6-050	antisense	ATTGTCAGTGAAGACCGGTG	3961-3980
HCV-6-051	sense	TGAACATCATGTGGTGGAAC	2860-2879
HCV-6-052	antisense	GCAAGCGTTGCAGCCACAGA	4110-4129
HCV-6-053	sense	AGGCCATATTGCTGGCATTA	1788-1807
HCV-6-054	antisense	GACATCGGTGCCAGATGGTCAT	4859-4880
HCV-6-055	sense	AGGTCTCCCCACTTACACCT	1907-1926
HCV-6-056	antisense	ATGTAGGTGCCAGTCCAAGC	3168-3187
HCV-6-051	sense	TGAACATCATGTGGTGGAAC	2860-2879
HCV-6-057	antisense	GCTGACATACAGGTCATGATG	5273-5293
HCV-6-049	sense	AAGTGTGGGTCCCGAGTATG	2914-2933
HCV-6-058	antisense	TTCCACATAGTGTCCCATGAGGGA	5144-5167
HCV-6-059	sense	TTGCCAGCTCTCCTGTCACCG	5988-6008
HCV-6-060	antisense	TTGTAGTCCGGCCKCGCCCAG	7205-7225
HCV-6-061	sense	GCACTATGTGCCTGAGACTGA	6137-6157
HCV-6-062	antisense	TTTGTTCTCRGACTCGACCC	7069-7088
HCV-6-063	sense	GTGCTSACGTCCATGCTCAC	6849-6868
HCV-6-064	antisense	TTGTAGTCCGGCCKCGCCCAG	7205-7225
HCV-6-065	sense	ACATCACYGCAGAGACGGCG	6880-6899
HCV-6-066	antisense	TTTGTTCTCRGACTCGACCC	7069-7088
HCV-6-067	sense	AGTACGCTAAAAGGGTCGTCG	3816-3836
HCV-6-068	antisense	GTTAGGCAGTATGCCGCTAGC	5348-5368
HCV-6-069	sense	CATGCAGTCGGGCTCTTCCG	3864-3883
HCV-6-070	antisense	CCAAGTGCTGGTGATGACTTC	5301-5321
HCV-6-071	sense	GTCGCTTTCTATCGGGGCGT	4584-4603
HCV-6-068	antisense	GTTAGGCAGTATGCCGCTAGC	5348-5368
HCV-6-069	sense	TGGCAGTTATTCCAACTGCAG	4609-4629
HCV-6-070	antisense	CCAAGTGCTGGTGATGACTTC	5301-5321
HCV-6-071	sense	AGTACGCTAAAAGGGTCGTCG	3816-3836
HCV-6-072	antisense	TCCTGTGGAACTGTGGTTGTC	4760-4780
HCV-6-073	sense	CATGCAGTCGGGCTCTTCCG	3864-3883
HCV-6-074	antisense	ATGGTGAAAGTGGGGTCTAGG	4736-4756
HCV-6-075	sense	CCTTCCTTGAAGGCGACGTGC	6966-6986
HCV-6-076	antisense	AACACCTCATTYTTRGCCAT	8028-8047
HCV-6-077	sense	CTGAGCTACTTGAAGCTAACC	7012-7032
HCV-6-078	antisense	ATGGTWGTAGGWATWGGTGT	8007-8026
HCV-6-079	sense	GAATGCAGATGTTATACCTG	3746-3765
HCV-6-080	antisense	GGTCGGACCTGTTAACATGG	5188-5207
HCV-6-081	sense	TGTGCCCTTCGGGGCATGCA	3850-3869
HCV-6-082	antisense	TCCACATAGTGTCCCATGAGG	5146-5166
HCV-6-083	sense	CTCCAACGGCTGAAGCCCATG	5172-5192
HCV-6-084	antisense	TTGACAGGTCCGACCCCCTT	5193-5212
HCV-6-085	sense	CAACCAGGAAGTAGCGCATGG	6252-6272
HCV-6-086	antisense	GTCTCCGTTAATCCACTCGTG	6222-6242
HCV-6-075	sense	CCTTCCTTGAAGGCGACGTGC	6966-6986
HCV-6-087	antisense	GTCATCAGTACCATGCGTACC	8873-8893
HCV-6-077	sense	CTGAGCTACTTGAAGCTAACC	7012-7032
HCV-6-088	antisense	ATCGTGGGCGCGTACATGATGA	8848-8869
HCV-6-089	sense	ATCCAAAGACTCCACGGAATG	9000-9020
HCV-6-090	antisense	AAAAAAAAAAAAAGTGGAGTGT	9414-9434
HCV-6-091	sense	TTTCACTCCATGGTTACTCGC	9028-9048
HCV-6-092	antisense	GTGTTATTTCTCCTCCCCGC	9390-9409
HCV-6-093	sense	CGGCAAGTACCTCTTCAATTG	9176-9196
HCV-6-094	antisense	AAAAAAAAAAAAAAAAAAAAAAAAAA	9419-9444
HCV-6-095	sense	GTGAGGACGAAGCTCAAACT	9201-9220
HCV-6-094	antisense	AAAAAAAAAAAAAAAAAAAAAAAAAA	9419-9444
HCV-6-096	sense	ACNAAGCTMAAACTCACTCC	9207-9226
HCV-6-097	antisense	AAAAAAAAAAAAAAAAAAAAAAAAAAAAAA	9415-9444
HCV-6-098	sense	TGGGSTTYKCSTATGAYACYCGMTGYTTTGA	8257-8287
HCV-6-099	antisense	AGTAGGAGTAGGCACAGGAGTA	9328-9349
HCV-6-100	sense	TATGATACCCGCTGCTTTGACTCCAC	8268-8293
HCV-6-101	antisense	GACACGCTGTGAAATATGTCC	9287-9307
HCV-6-093	sense	CGGCAAGTACCTCTTCAATTG	9176-9196
HCV-6-102	antisense	CTGATCTAGAGGTACCGGATCCAAAAAAAAAAAAAAAAAA	9247-9466
HCV-6-095	sense	GTGAGGACGAAGCTCAAACT	9201-9220
HCV-6-102	antisense	CTGATCTAGAGGTACCGGATCCAAAAAAAAAAAAAAAAAA	9247-9466
